# Quantitative Trait Locus (QTL) meta-analysis and comparative genomics for candidate gene prediction in perennial ryegrass (*Lolium perenne* L.)

**DOI:** 10.1186/1471-2156-13-101

**Published:** 2012-11-08

**Authors:** Hiroshi Shinozuka, Noel OI Cogan, German C Spangenberg, John W Forster

**Affiliations:** 1 Department of Primary Industries, Biosciences Research Division, Victorian AgriBiosciences Centre, 1 Park Drive, La Trobe University Research and Development Park, Bundoora, Victoria 3083, Australia; 2 Dairy Futures Cooperative Research Centre, Bundoora, Australia; 3 La Trobe University, Bundoora, Victoria 3086, Australia

**Keywords:** Quantitative variation, Pasture grass, BioMercator software, Comparative genetics, Genetic map, Molecular breeding

## Abstract

**Background:**

In crop species, QTL analysis is commonly used for identification of factors contributing to variation of agronomically important traits. As an important pasture species, a large number of QTLs have been reported for perennial ryegrass based on analysis of biparental mapping populations. Further characterisation of those QTLs is, however, essential for utilisation in varietal improvement programs.

**Results:**

A bibliographic survey of perennial ryegrass trait-dissection studies identified a total of 560 QTLs from previously published papers, of which 189, 270 and 101 were classified as morphology-, physiology- and resistance/tolerance-related loci, respectively. The collected dataset permitted a subsequent meta-QTL study and implementation of a cross-species candidate gene identification approach. A meta-QTL analysis based on use of the BioMercator software was performed to identify two consensus regions for pathogen resistance traits. Genes that are candidates for causal polymorphism underpinning perennial ryegrass QTLs were identified through *in silico* comparative mapping using rice databases, and 7 genes were assigned to the p150/112 reference map. Markers linked to the *Lp*DGL1, *Lp*Ph1 and *Lp*PIPK1 genes were located close to plant size, leaf extension time and heading date-related QTLs, respectively, suggesting that these genes may be functionally associated with important agronomic traits in perennial ryegrass.

**Conclusions:**

Functional markers are valuable for QTL meta-analysis and comparative genomics. Enrichment of such genetic markers may permit further detailed characterisation of QTLs. The outcomes of QTL meta-analysis and comparative genomics studies may be useful for accelerated development of novel perennial ryegrass cultivars with desirable traits.

## Background

Perennial ryegrass is a native species of Europe, temperate Asia and North Africa and is widely cultivated in temperate regions as a pasture crop [1,2]. This obligate outbreeding diploid species (2n = 2x = 14) is classified within the Pooideae sub-family of the Poaceae (grass and cereal) family [3]. The Pooideae sub-family contains a broad range of important cereal and forage crop species; hexaploid wheat (*Triticum aestivum* L.) and barley (*Hordeum vulgare* L.) are taxonomically classified into the Triticeae tribe; oat (*Avena sativa* L.) is a representative member of the Aveneae tribe; and perennial ryegrass, tall fescue (*Festuca arundinacea* Schreb.) and meadow fescue (*F. pratensis* Huds.) are included in the Poeae tribe [4]. Genome analysis studies have suggested that species in the Pooideae sub-family share a similar chromosomal structure, having been derived from a common ancestor with 7 chromosome pairs [5].

In crop species, agronomically important traits, such as grain number and salinity stress tolerance, are governed by multiple loci with relatively small individual effects, which are known as QTLs [6]. Favorable alleles of QTLs are able to be efficiently introduced into elite cultivars through marker-assisted selection (MAS) technology to generate new varieties with enhanced yield performance and adaptability to environmental conditions [7-9]. During the last decade, a number of molecular genetic studies of perennial ryegrass have been conducted to reveal the genetic basis of herbage quality and productivity. A one-way pseudo-testcross population, designated p150/112, was established through crossing between a multiple heterozygous parent of complex descent (C3) and a doubled haploid parent [10,11]. Using this population, the first comprehensive genetic linkage map for perennial ryegrass was constructed using simple sequence repeat (SSR), amplified fragment length polymorphism (AFLP) and restriction fragment length polymorphism (RFLP) markers [11,12]. The seven linkage groups (LGs) were numbered in accordance with conserved synteny with the genetic maps of the Triticeae cereal species [12]. The p150/112 population was also subjected to QTL analyses for plant architecture traits, herbage yield and quality characters, cold tolerance, heading date variation and seed production [13-15]. Following effective use of the p150/112 population, a number of successor mapping populations were developed for QTL identification across a range of common and additional traits [16-20].

The process of QTL meta-analysis was proposed as a means to identify consensus loci reported in numerous distinct studies [21]. The BioMercator software was designed to perform meta-QTL (MQTL) analysis using published data [22]. MQTL analysis has been achieved with the BioMercator software for a wide range of crop species, such as rice (*Oryza sativa* L.), wheat and soybean (*Glycine max* L.) [23-25]. Despite complexities of genome structure, 5 and 12 relatively large MQTLs were successfully identified in soybean and hexaploid wheat, respectively [24,25]. Due to properties of stability under different environmental and genetic backgrounds, such meta-QTLs (MQTLs) are likely to be of particularly high value for breeding activities [24,26]. The MQTLs identified in the previous studies provide primary targets for fine-structure mapping and gene identification activities [24,25]. As the number of published trait-dissection studies has increased for perennial ryegrass, so this species has become a viable target for QTL meta-analysis.

Macrosyntenic relationships of genome structure between perennial ryegrass and taxonomically related cereal species, such as rice, wheat and oat have been demonstrated through cross-species mapping of functional genetic markers [12,27]. By permitting transfer of knowledge from the related species, such colinearity has been used for identification of candidate genes that potentially underpin QTL-containing regions. For instance, co-locations were demonstrated between candidate ortholoci of rice heading date control genes and QTLs for flowering time variation in perennial ryegrass, suggesting functional similarity of these genes between the two species [15,28,29]. Similarly, the *Lp*ABCG5 gene was proposed to contribute to a plant architecture QTL effect in perennial ryegrass, based on a comparative genomics approach between related species, including rice [30]. The value of such an approach for gene identification is currently higher than for map-based cloning strategies in species such perennial ryegrass, as compared to inbreeding species, due to an obligate outbreeding reproductive habit and relatively large genome size [31].

In this study, a bibliographic survey of QTLs that were identified through use of perennial ryegrass-based genetic mapping populations during the last decade is presented. An MQTL analysis for selected loci across a range of functional categories, in concert with comparative analysis with rice QTL databases, was performed. Putative candidate genes were identified and subjected to a genetic linkage analysis with the p150/112 reference mapping population, providing the basis for assessment of QTL co-location in present and future studies.

## Methods

### Bibliographic survey and QTL categorisation

In order to collate information on perennial ryegrass QTLs, 23 previously published studies were identified, details of which are summarised in Additional file 1[2,13-15,17,18,20,29,32-46]. Prefixes relating to nomenclature of the genetic marker classes are explained in Additional file 2[47]. QTLs were categorised into 3 functionally related groups (morphology, physiology and resistance/tolerance), and 6–10 sub-groups (e.g. leaf/pseudostem), depending on trait features as previously described in an equivalent study of rice [48].

### MQTL analysis for pathogen resistance QTLs

Identification of consensus QTLs was performed using the BioMercator software [22]. The p150/112, NA_6_ and AU_6_ maps were initially integrated and then aligned with the WSCF_2_, MFA, MFB and SB2 x TC1 genetic maps [11,12,17,33,34,36,41,43,44]. Locations of QTLs for pathogen resistance were extrapolated onto the consensus map on the basis of common genetic marker positions. Co-location of QTLs was determined on the basis of the Akaike’s information criterion (AIC), and the best fit model was selected for MQTL prediction.

### In silico *comparative genomic analysis*

DNA sequence information was obtained from the NCBI ( http://www.ncbi.nlm.nih.gov/), GrainGene ( http://www.gramene.org/) and Phytozome ( http://www.phytozome.net/) databases. The physical locations of orthologous genes were identified using the Phytozome database. Candidate genes for perennial ryegrass QTLs were identified using the Q-TARO [48] ( http://qtaro.abr.affrc.go.jp/) and GRAMENE QTL ( http://www.gramene.org/qtl/) databases and by bibliographic search of the NCBI database.

### Genetic marker development and linkage analysis

The p150/112 one-way pseudo-testcross mapping population was generated through crossing of a multiply heterozygous genotype to an artificially generated (doubled haploid) homozygote. In this mating design, single nucleotide polymorphisms (SNPs) exhibit segregation patterns of the type AB x AA or AB x CC [10-12]. As a bin-mapping population sub-set, 46 genotypes, which represent individuals with maximal genetic recombination, were selected. Candidate orthologues for rice genes were identified through local BLAST searches. Locus-specific PCR primers were designed using the Sequencher^TM^ software version 4.7 for windows (Genecodes) and Oligo Calc program [49]. PCR amplification was performed using Immolase^TM^ DNA polymerase (BIOLINE, London UK) following the product instructions. The PCR amplification was examined on a 2.0% (w/v) agarose gel with 0.5 x SYBR® Safe DNA gel staining (Invitrogen). The PCR products were treated with exonuclease I (0.5 U) and shrimp alkaline phosphatase (SAP; 0.5 U) at 37°C for 60 minutes, and enzymes were then deactivated by heat treatment at 85°C for 20 minutes. Sequencing analysis was performed with the BigDye^TM^ terminator chemistry (Applied Biosystems, at present Applera, Foster City California USA), following the manufacturer’s instructions, and the resulting products were analysed on the ABI 3730xl Prism sequencer (Applied Biosystems). SNPs in the targeted sequence were identified using the Sequencher^TM^ software, and genotyping data were scored. A genetic linkage map was constructed using the JoinMAP 3.0 application [50].

## Results

### Assembly of information on previously identified perennial ryegrass QTLs

A total of 560 QTLs were described in previously published studies, of which 149 were identified from analysis of perennial ryegrass x Italian ryegrass interspecific hybrid populations [33,34,41,45,46]. Totals of 189 (34%), 270 (48%) and 101 (18%) were categorized as morphology-, physiology- and resistance/tolerance-related QTLs, respectively (Additional file 1: Table S1). Of the 6 sub-classes of morphological QTLs, the proportion of leaf/pseudostem-related loci was 36% (68 QTLs), followed by panicle/flower (24%) and plant mass-related loci (23%). The most consistently reported physiological QTLs were fibre content-related loci. In the resistance/tolerance-related QTL group, 27 hydrate/dehydrate stress, 26 cold stress and 27 crown rust resistance-related loci were found. Totals of 106 and 100 QTLs were located on LGs 1 and 4, respectively, while relatively smaller number of loci were identified on LGs 5 (49 QTLs) and 6 (46 QTLs). This trend was also observed within each trait class (Table 1; Additional file 3).

**Table 1:** Perennial ryegrass QTLs classified by trait class

**Trait class**	**Trait sub-class**	**Number of QTL**							
		**LG1**	**LG2**	**LG3**	**LG4**	**LG5**	**LG6**	**LG7**	**Total**
Morphological	Plant mass	5	8	10	8	5	2	6	**44**
	Plant height/type	4	3	3	4	1	0	5	**20**
	Leaf/pseudostem	13	7	17	15	3	5	8	**68**
	Panicle/flower	5	11	7	13	4	3	3	**46**
	Root	2	1	1	2	0	0	0	**6**
	Seed	2	0	1	1	0	0	1	**5**
Physiological	Heading date	1	4	4	8	2	3	9	**31**
	Growth	6	4	9	7	5	6	3	**40**
	Fertility	3	2	0	1	0	0	3	**9**
	Protein content	3	8	3	3	2	0	1	**20**
	Carbohydrate content	6	3	3	1	2	7	3	**25**
	Fibre content	14	9	6	0	5	10	16	**60**
	Alkaloid	8	3	0	10	2	0	4	**27**
	Other/unknown content	16	13	2	11	0	0	8	**50**
	Digestibility	0	0	3	1	0	0	2	**6**
	Other	0	0	0	2	0	0	0	**2**
Resistance-Tolerance	Hydrate/dehydrate stress	8	0	8	2	5	4	0	**27**
	Cold stress	2	3	5	6	10	0	0	**26**
	Crown rust resistance	4	5	4	4	3	2	5	**27**
	Stem rust resistance	3	0	0	0	0	2	4	**9**
	Grey leaf spot resistance	0	1	3	1	0	2	0	**7**
	Powdery mildew resistance	0	0	1	0	0	0	2	**3**
	Wiltiness	1	1	0	0	0	0	0	**2**
**Total**		**106**	**86**	**90**	**100**	**49**	**46**	**83**	**560**

**Figure 1:**
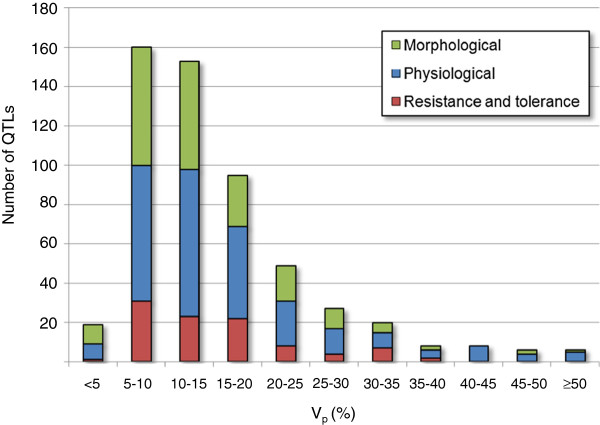
**Distribution of V**_**p**_**values for perennial ryegrass QTLs.**

The 560 QTLs were classified depending on percentages of phenotypic variances explained (V_p_) (Figure 1). About 60% of the QTLs displayed a V_p_ value of less than 15%, while 28 QTLs (5%) explained more than 40% of the phenotypic variance. The average and median values of V_p_ were 15.8% and 13.1%, respectively.

### MQTL analysis

A consensus map was constructed based a combination of the p150/112, AU_6_, NA_6_, WSCF_2_, MFA, MFB and SB2 x TC1 maps. Due to insufficiency of common genetic markers, map melding was not performed with linkage maps from the other published studies. Pathogen resistance QTLs were subjected to analysis performed with the BioMercator software. Two MQTLs, designated mqResis-2 and mqResis-6, were identified on LGs 2 and 6 of the consensus map, respectively (Table 2). The mqResis-2 MQTL contained a grey leaf spot resistance and three crown rust resistance QTLs, while the mqResis-6 MQTL was a consensus of two grey leaf spot resistance loci and a crown rust resistance locus.

**Table 2:** Characteristics of pathogen resistance MQTLs

**MQTL**	**Location**	**Composing QTL**	**QTL feature**	**Vp**	**Reference**	**Flanking functional marker**	
**Name**		**Name**				**Name**	**Position**
mqResis-2	LG2; 25.93 cM	qCrownrust04_WI1_MFA2	Crown rust resistance	13.1	[41]	Lpest0222-472	LG2; 22.4 cM
		qCrownrust04_WI2_MFA2	Crown rust resistance	11.4	[41]	BCD1184	LG2; 25.7 cM
		qCrownrust05_WI1_MFA2	Crown rust resistance	15.3	[41]		
		qGLS6082gc_MFA_2	Grey leaf spot resistance	8.9	[34]		
mqResis-6	LG6; 101.83 cM	qCrown_rust_PS_2005_NA6_6	Crown rust resistance	5.9	[17]	CDO497	LG6; 100.2 cM
		qGLSGG9gc_1_MFA_6	Grey leaf spot resistance	9.5	[34]	RZ273	LG6; 101.9 cM
		qGLSGG9gc_2_MFA_6	Grey leaf spot resistance	9.2	[34]		

### Cross-species candidate gene identification

Information required for the comparative candidate gene identification approach was obtained through the bibliographic survey of QTLs ( Additional file 1). Of the 560 QTLs, putative functional markers were identified in the flanking regions of 265 loci. For 212 QTLs, orthologous regions in the rice genome were predicted using sequence information from flanking genetic markers. The candidate regions for 19 QTLs were not, however, located on orthologous chromosomes. For 45 perennial ryegrass QTLs, equivalent QTLs in orthologous regions of the rice genome were identified. A total of 10 rice candidate genes, for which ortholoci may contribute to perennial ryegrass QTL variation, were tentatively recognised (Table 3) [51-60].

**Table 3:** Genetic locations of candidate gene-derived markers and QTLs on the p150/112 reference genetic map. Chr denotes chromosome

**Candidate rice ortholocus**		**Reference**	**Prerennial ryegrass QTL**	**LG**	**Reference**	**Anchor marker**	
**Gene**	**Physical location (Chr.: Mb)**		**QTL**			**Marker**	Physical location^*a^**(Chr.: Mb)**
*EUI1*	Chr. 5: 23.7 Mb	[51]	qPlantheight_C3_1	1	[13]	PSR162	Chr. 5: 25.5 Mb
*PDT*	Chr. 7: 30 Mb	[52]	qCP-Sep-03-f2	2	[46]	CDO418	Chr. 7: 27.6 Mb
			qCP-Sep-03-m2	2	[46]	CDO59	Chr. 7: 26.4 Mb
			qCP-04-m2	2	[46]	RZ395	Chr. 7: 24.6 Mb
			qCP-04-f2	2	[46]	CDO405	Chr. 7: 27.6 Mb
			qCP-su-gh-01_2	2	[14]	CDO405	Chr. 7: 27.6 Mb
*OsGA3ox2*	Chr. 1: 4 Mb	[53]	qFG-04-f3.2	3	[45]	CDO460	Chr. 1: 1.4 Mb
DGL1^*b^	Chr. 1: 28.2 Mb	[54]	qLeafwidth_C3_3	3	[13]	CDO345	Chr. 1: 37.9 Mb
GA20ox-2^*b^	Chr. 1: 30 Mb	[55]	qPlantheight_C3_3	3	[13]	CDO345	Chr. 1: 37.9 Mb
Ph1^*b^	Chr. 1: 38.3 Mb	[56]	qTillersize_C3_3	3	[13]	CDO345	Chr. 1: 37.9 Mb
			qLET_WSCF2_3	3	[44]	CDO345	Chr. 1: 37.9 Mb
			qFG-03-m3	3	[45]	CDO345	Chr. 1: 37.9 Mb
*Pi37*	Chr. 1: 33.1 Mb	[57]	qPMR1_INF1_VrnA	3	[40]	LRGA4	Chr. 1:20.6 Mb
			qCrownrust04_WI2_MFB3	3	[41]	RZ444	Chr. 1:29.7 Mb
*OsPIPK*1	Chr. 3: 28.2 Mb	[58]	qHD_PxA_4	4	[29]	CDO795	Chr. 3: 30.1 Mb
			qHD_WSC_4	4	[15]	CDO795	Chr. 3: 30.1 Mb
			qHD_C3_4	4	[15]	CDO795	Chr. 3: 30.1 Mb
*Pib*	Chr. 2: 35.1 Mb	[59]	qGLSGG9gc_1_MFA_6	6	[34]	CDO686	Chr. 2: 31.8 Mb
			qGLSGG9gc_2_MFA_6	6	[34]	CDO686	Chr. 2: 31.8 Mb
*D3*	Chr. 6: 2.8 Mb	[60]	qLL_p150/112_7	7	[15]	Hd3a	Chr. 6: 2.9 Mb
			qLL_WSC_7	7	[15]	Hd3a	Chr. 6: 2.9 Mb

*^a^ Location of candidate orthologue in rice.

*^b^ Candidate genes for Leafwidth_C3_3, Plantheight_C3_3, Tillersize_C3_3, LET_WSCF2_3 and qFG-03-m3.

A total of 6 candidate genes were identified for plant morphogenesis traits. The PSR162-derived marker was located within the confidence interval containing a plant height QTL (Plantheight_C3_1) on perennial ryegrass LG1. A putative ortholocus for PSR162 was identified at the 25.5 Mb location on rice chromosome 5, and the rice *EUI1* (elongated uppermost internode1) gene, which is responsible for control of internode length, was found at the closely adjacent 23.7 Mb position of the same chromosome.

A total of 5 herbage yield-related QTLs (Leafwidth_C3_3, Plantheight_C3_3, Tillersize_C3_3, LET_WSCF2_3 and qFG-03-m3) were observed to be linked to the CDO345-derived marker on perennial ryegrass LG3. A candidate CDO345 ortholocus was identified at the 37.9 Mb position on rice chromosome 1, and 3 plant size-related genes (*DGL1*, *GA20ox-2* and *Ph1*) were observed in the 28.2-38.3 Mb region of the same chromosome.

Leaf length QTLs (qLL_WSC_7 and qLL_p150/112_7) were located on perennial ryegrass LG7, of which the maximum LOD score was observed at the Hd3a locus. On rice chromosome 6, the *D3* gene (2.7 Mb) was located closely adjacent to the rice Hd3a gene (2.9 Mb).

A fall growth QTL, qFG-04-f3.2, was closely associated with the CDO460-derived marker. A putative CDO460 ortholocus was identified at the 1.4 Mb position of rice chromosome 1, relatively close (at the 4 Mb coordinate) to the dwarf growth locus *OsGA3ox2*.

A single candidate gene was identified for nutritive quality traits. Crude protein concentration QTLs (qCP-Sep-03-f2, qCP-Sep-03-m2, qCP-04-m2, qCP-04-f2 and qCP-su-gh-01_2) were identified on perennial ryegrass LG2, linked to the CDO385, CDO418, CDO59, RZ395, CDO1376 or CDO405-derived markers, for which candidate orthologues were located in the 18.5-27.6 Mb interval of rice chromosome 7. A rice phenylalanine biosynthesis gene, *PDT*, was identified at the 30.0 Mb position of rice chromosome 7. The role of *PDT* is to control accumulation level of the amino acids phenylalanine and tryptophan.

A single candidate gene was identified for reproductive development traits. Maximum LOD values for heading date variation QTLs (qHD_PxA_4, qHD_WSC_4 and qHD_C3_4) were identified close to the CDO795-derived marker on perennial ryegrass LG4. A heading date locus (*OsPIPK*1) was located at the 28.2 Mb position of rice chromosome 3, close to the predicted CDO795 ortholocus (23.1 Mb).

A total of three candidate genes were identified for pathogen resistance traits. QTLs for resistance to powdery mildew (PMR1_INF1_VrnA) and crown rust (qCrownrust04_WI2_MFB3) pathogens were located on perennial ryegrass LG3, in linkage to the LRGA4 and RZ444 loci, respectively. The *Pi37* blast resistance locus is located at the 33.1 Mb position on rice chromosome 1, relatively close to putative ortholoci for LRGA4 (20.6 Mb) and RZ444 (35.9 Mb).

The CDO686-derived marker was located in linkage with grey leaf spot resistance QTLs (qGLSGG9gc_1_MFA_6 and qGLSGG9gc_2_MFA_6) on perennial ryegrass LG6. A putative CDO686 ortholocus is located at the 31.8 Mb position of rice chromosome 2, and the rice *Pib* locus, which is responsible for resistance to rice blast, was observed at the 35.1 Mb coordinate on the same chromosome.

MQTLs were also subjected to the cross-species mapping approach. Sequences orthologous to BCD1184 and Lpest0222, which were located close to mqResis-2, were identified on rice chromosome 2 at the 29.4 Mb and 30.1 Mb positions. A candidate orthologous region for mqResis-6 was identified on rice chromosome 7, through identification of putative CDO497 and RZ273 ortholoci at the 23.7 Mb and 30.7 Mb coordinates, respectively. No equivalent rice QTL was, however, identified for the MQTLs.

### Genetic mapping of candidate genes

**Figure 2:**
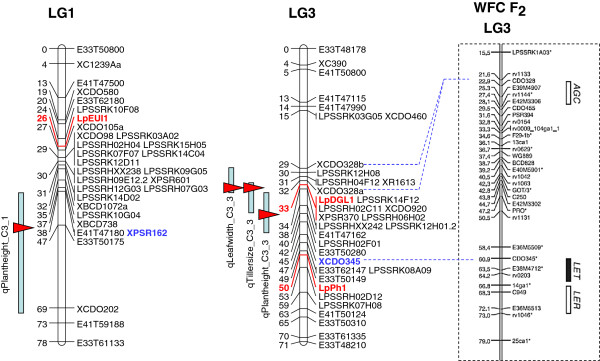
**Genetic locations of candidate gene-derived markers and QTLs on LGs 1 and 3 of the p150/112 reference genetic map.** Genetic distance (cM) is shown on the left side of the genetic map. Candidate gene-derived markers are represented in red. The positions of the QTL interval and maximum LOD value are shown on the left side of linkage map with the light blue line and red triangle, respectively. The functional markers used for comparative analysis are represented in blue. The LG3 map constructed with the WFC F_2_ population and QTL intervals for autumn growth (*AGC*), leaf extension time (*LET*) and leaf extension rate (*LER*) is shown on the right side of the LG3 map of the p150/112 map (cited from [44]), and corresponding makers are connected with broken blue lines.

**Figure 3:**
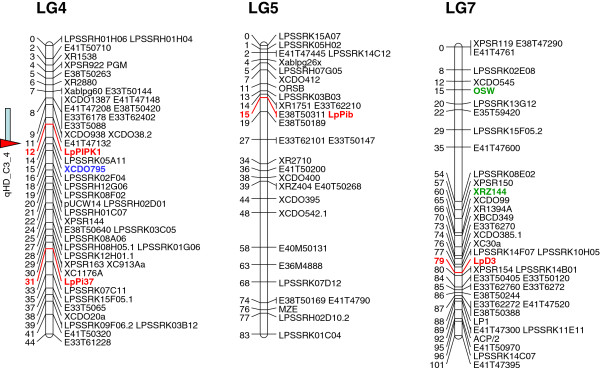
**Genetic locations of candidate gene-derived markers and QTLs on LGs 4, 5 and 7 of the p150/112 reference genetic map.** Details are as described for Figure 2. The OSW and RZ144 sequence-related markers on LG7 are shown in green, close to which leaf length QTLs were identified [15].

PCR primers for the putative perennial ryegrass ortholoci of the 10 rice candidate genes were designed, and PCR fragments from 9 of the target genes were obtained from the p150/112 C3 (heterozygous) parent (Table 4). Successful amplification was not observed when the *OsGA3ox2* ortholocus-directed primer pair was used. Direct sequencing analysis identified SNPs in 7 of 9 amplicons, DNA sequence polymorphism being absent from amplicons corresponding to the *GA20ox-2* and *PDT* genes. The p150/112 bin mapping set was genotyped to obtain successful sequencing data from 30–46 individuals. Selected SNP loci from the *EUI1*, *DGL1*, *Ph1*, *OsPIPK*1 and *D3* gene orthologues were assigned to locations on those LGs that were anticipated on the basis of conserved synteny, while those in *Pi37* and *Pbi*-like sequences were assigned to LGs 4 and 5, respectively (Figures 2 and 3).

**Table 4:** PCR primer sequence for candidate QTL-related gene and SNP type used for genotypic analysis

**Candidate genes**	**Primer sequence (5'→3')**	**SNP**
*Lp*EUI	f: ACG TAC CTG TAC TGG CTG	C/G
	r: TTG CAG TTG TCC ACC ACG AA	
*Lp*PDT	f: GCA GAA CAA AAA CTC CAA GA	n.a. *a
	r: TTG GAT CAG CCA TAG ACG CC	
*Lp*GA3ox2	f: CGC GCT ACT TCG ACT TCC	n.a. *b
	r: GAA GAA GGA GGA GAT GGG C	
*Lp*DGL1	f: GTT AAC ATT GAT GAA GTT GC	A/G
	r: ACA CTC TTC TGG ACC TTG GC	
*Lp*GA20	f: GGG TGT ACC AGG AGT ACT G	n.a. *a
	r: TTA CCA TGA AGG TGT CGC CG	
*Lp*Ph1	f: GCA TTA ATG ATG AAT GGG CT	A/G
	r: CAT CCA CAC CAG TTA TTC TC	
*Lp*Pi37	f: CCA GCG GAT ATG CGC AAT CT	C/T
	r: CAA ATG CTC TCG GCT GAA GG	
*Lp*PIPK1	f: GGC CCT TGT AAA TAG TCT CC	G/T
	r: CCC TTG ACT GTA ATT GGC TC	
*Lp*Pib	f: TCA CGG ATG AGA TCA TGG AC	A/T
	r: CTG AAG AAG TGT GAT GGA CT	
*Lp*D3	f: CCA AGA TGA AAT TGG ACC TC	A/G
	r: CTG CAT GTC CCG CAA GTT TG	
*a no SNP was identified in PCR amplicon.		
*b PCR amplicons were not obtained.		

## Discussion

### QTL meta-analysis in perennial ryegrass

As a pasture crop species, a predominant focus on vegetative yield-related characters has been observed during trait-dissection studies of perennial ryegrass, leading to identification of a large number of leaf/pseudostem and plant mass-related QTLs (Table 1). In contrast, as a grain crop, panicle/flower and seed-related traits have received more attention in rice QTL identification activities [48]. In the present study, lower QTL numbers were identified on LGs 5 and 6. In comparison, a meta-study for hexaploid wheat grain yield-related QTLs identified relatively smaller numbers of QTLs were identified on the homoeologous 5, 6 and 7 groups of chromosomes [24], which exhibit extensive macrosynteny with perennial ryegrass LGs 5, 6 and 7 [12]. Hence, despite divergent trait-specific biases between perennial ryegrass and hexaploid wheat, a similar chromosomal distribution pattern of QTLs was exhibited (Table 1). In a previous study of grain yield under drought stress conditions, conservation of QTL locations between different Poaceae species was observed [26]. As perennial ryegrass and wheat are relatively closely related within the cool-season grasses, the similarity of QTL distribution patterns between these two species suggests that conserved regions corresponding to wheat homoeologous chromosomes 5, 6 and 7 show lower importance than others for agronomic traits, including both vegetative and seed yield characters. Due to a large genome size, the perennial ryegrass genome has not yet been completely sequenced and assembled. Full assembly of genome sequence information from chromosomes that are rich in important QTLs may be more valuable, and should perhaps be prioritised, in comparison to that from other chromosomes.

The bibliographic survey identified putatively conservation of QTL locations under different environmental and across different genetic backgrounds. Plant height QTLs on LG1 were reported in three distinct studies, and heading date QTLs on LGs 4 and 7 were identified with various parental combinations at multiple geographic locations, although further analysis is required to determine whether the common QTLs are controlled by identical genetic factors [13,15,18,29,32,42]. The two pathogen resistance MQTLs are also putatively conserved under multiple environmental conditions and genetic backgrounds. Conversely, evidence was also obtained for a relatively large number of QTLs that are either genotype- or environment-specific. QTL analysis studies with two-way pseudo-testcross populations have demonstrated the presence of QTLs only on single parental genetic maps for traits measured under identical environmental conditions [2,17,32]. Several studies also subjected single populations to QTL analysis under various environmental conditions, and reported environment-specific QTLs [33,42,45,46]. The p150/112 mapping population was developed for the activities of the International *Lolium* Genome Initiative (ILGI) and was subjected to QTL analysis for traits such as leaf length, leaf width, and variation for heading date in both Japan and the UK, identifying unique QTLs at the two geographic locations [13,15]. Leaf length and width QTLs were identified on LGs 5 and 3, respectively under Japanese conditions, while QTLs for both traits were found on LG7 in the UK-based trial. Only a single heading date QTL on LG4 was detected in Japan, while two QTLs on LGs 4 and 7 were found in the UK, probably associated with vernalisation genes (*Vrn-1* and *Hd3a* orthologues, respectively). These results suggest that although stable QTLs may be detected under different environmental and genetic backgrounds, QTL identification largely depends on both genetic and environmental factors in perennial ryegrass.

The frequency distribution of V_p_ demonstrated in this study (Figure 1) was also similar to that obtained from a previous study in rice, in which the mean V_p_ value was calculated to be c. 13%, based on a sample of 231 QTLs [61]. In both studies, although the distribution range was skewed towards lower V_p_ values, a considerably small number of QTLs were classed in the 0-5% category. The probable presence of loci of minor effect, which could be excluded from identification due to the requirement for threshold LOD values for QTL detection, was also described for the rice study, and such minor undetected QTLs are also likely to be present in perennial ryegrass. Although F_2_ and BC_1_ genetic mapping populations have been generally employed for rice, construction of perennial ryegrass linkage maps has been commonly based on use of one-way and two-way pseudo-testcross strategies, due to the effect of an outbreeding reproductive habit. These crossing formats may not achieve such precise estimation of QTL effects as the F_2_ and BC_1_ designs, due to complexity of genetic background [62]. Despite this difference, the distribution patterns of V_p_ values were largely similar between the two species.

Due to the relatively small sizes of discovery populations (typically in the range from 100–200 genotypes) that have been used for trait-dissection in perennial ryegrass, the magnitudes of QTL effects have probably been over-estimated. Several studies have identified failures to deliver anticipated genetic gains through marker-assisted QTL selection, due apparently to both over-estimation and imprecise estimates of location [63,64]. The basis of these problems has been extensively discussed, and has in most cases been attributed to the influence of experimental population size, the so-called Beavis effect [65-67]. QTL identification in progressively larger population sets, up to 500–1000 individuals, has been theoretically and empirically demonstrated to enhance the accuracy of QTL effect measurement. Alternatively, more accurate estimates of locus-specific effect are likely to derive from implementation of genome-wide association studies (GWAS). For example, a GWAS for 14 agronomic traits in rice identified six characters associated with colours, grain quality and grain width that exhibited a small number of significant loci with large effects, while the remaining traits were influenced by multiple loci with relatively small effects [68]. Equivalent studies in perennial ryegrass might be anticipated to generate similar results.

The BioMercator software assisted the melding of linkage maps resulting from distinct studies. This process, however, was not fully accomplished in the present study, except for the p150/112, AU_6_, NA_6_, WSCF_2_, MFA, MFB and SB2 x TC1 maps, due to insufficiency of common genetic markers. In previous studies, non-functional DNA-based markers, such as genomic DNA-derived SSR, AFLP and restriction site-associated DNA (RAD) systems, were predominantly used [18,32,37]. Such assays are not ideally suited to comparative mapping studies, as multiple locus amplification is often observed for genomic DNA-derived SSR markers, and both AFLPs and RADs are more genotype-specific than functional markers [11,31,37]. Enrichment of functional markers is hence essential for a further meta-analysis. A recent study assigned over 700 gene-derived markers to perennial ryegrass LGs with public release of the corresponding information [69]. The outcomes may permit efficient functional marker enrichment in specific chromosomal regions of interest.

### Prediction of candidate gene status

Two putative MQTLs were identified for pathogen resistance (Table 2). Both mqResis-2 and mqResis-6 were identified as consensus loci containing both grey leaf spot and crown rust resistance QTLs, implying non-specific activities for several pathogens, rather than race-specific resistance QTLs. Through the process of genetic map alignment and MQTL analysis, additional functionally associated markers that are putatively linked to the QTLs were identified. Information from functional markers may support development of novel flanking DNA-based markers for a given target locus based on a comparative genetics approach, enabling candidate gene-based selection and association genetics studies [15,70]. Although further characterisation is required, both MQTLs and flanking functional markers may be useful for deployment in perennial ryegrass breeding.

Comparative analysis demonstrated close proximity between genetic markers related to the *DGL1*, *Ph1* and *OsPIPK*1 ortholoci and the corresponding perennial ryegrass QTLs. This observation suggests that the *DGL1* and *Ph1* ortholoci are related to, and may provide candidate genes for, the herbage yield-related QTLs on LG3. In a previous study, the CDO795-linked heading date QTLs were suggested to be equivalent to a rice heading date QTL, *dth3.3* (Gramene QTL Acc. ID AQFE011) [15,71]. The physical location of the *OsPIPK*1 gene was located in the candidate interval (5.7 Mb) of *dth3.3*. These results suggest that the perennial ryegrass *OsPIPK*1 ortholocus may be related to the heading date QTLs on LG4. For both yield and flowering time traits, plausible evidence for related candidate genes has been obtained.

In contrast, markers linked to the *EUI1* and *D3* ortholoci were located over 10 cM distant from the maximum LOD values for the target QTLs. In a wide range of plant species, genes causing variation in quantitative traits have been identified to be located within genetic distances of less than 3 cM from the LOD maximum location [72]. It seems, therefore, unlikely that *Lp*EUI1 and *Lp*D3 genes are plausible candidates for QTL function. For issues arise for candidate genes associated with disease resistance.

The *Pi37* and *Pbi* genes encode NBS-LRR proteins [51,59]. Molecular studies have shown a rapid evolutionary rate and limited cross-species synteny of NBS-LRR genes [51,59,73,74]. The comparative approach may not be so effective for such species-specific genes, due to unresolved paralogous relationships between species, and hence accounting for the failure of putative ortholoci to map in regions predicted on the basis of conserved synteny.

## Conclusion

In this study, meta-analysis of QTL architecture in perennial ryegrass has permitted evaluation of the range of typical genetic effects across a range of biological trait categories. Additionally, MQTL analysis identified two consensus QTLs for pathogen resistance, as well as putatively linked functional markers. Comparative genetics analysis for a sample of putative candidate genes revealed ortholoci of three rice genes that may plausibly be causally related to QTLs for correlated functions. Enrichment of functional markers may permit further Meta-analysis and comparative approach for those QTLs. Outcomes from those studies may be utilised in the MAS framework for varietal development of perennial ryegrass with desirable traits.

## Authors’ contributions

HS co-conceptualised the project, performed the bibliographic survey and experimental work, prepared the tables and figures and the primary drafts of the manuscript, and contributed to finalisation of the text and journal-specific formatting. NC co-conceptualised the project, provided perennial ryegrass sequence information and contributed to computational analysis. GS assisted the project leadership and co-developed the final draft of the manuscript. JF co-conceptualised the project, provided overall project leadership, and co-developed interim and final drafts of the manuscript. All authors have read and approved the final manuscript.

## Supplementary Material

Additional file 1 **Summary information on QTLs identified through use of perennial ryegrass-based genetic mapping populations.** QTLs are designated according to the following nomenclature: trait/date/condition abbreviation_experiment replication number/location_genetic map/population name_LG location_QTL identity (e.g. a or b) for the purpose of locus discrimination, as needed. For analysis type, IM, SIM, CIM, MQM and SMR stand for interval mapping, simple interval mapping, composite interval mapping, multiple QTL mapping and single-marker regression. When multiple parameters (e.g. SIM and CIM) are used for QTL detection, only QTLs identified with the representative parameter are shown. For population type, 1-way, 2-way and F_2_ stand for one-way pseudo-testcross population, two-way pseudo-testcross population and F_2_ genetic mapping population types. Click here for file

Additional file 2 Nomenclature of prefixes denoting classes of DNA-based marker. Click here for file

Additional file 3 Distribution of QTLs in each trait class on the seven perennial ryegrass LGs. Click here for file
