# Supplementary figures and images for: Quantitative Trait Locus (QTL) meta-analysis and comparative genomics for candidate gene prediction in perennial ryegrass (Lolium perenne L.)
Source: BMC Genet. 2012 Nov 8;13:101. doi: 10.1186/1471-2156-13-101 (PMC3532372; doi:10.1186/1471-2156-13-101)

## Slide 1
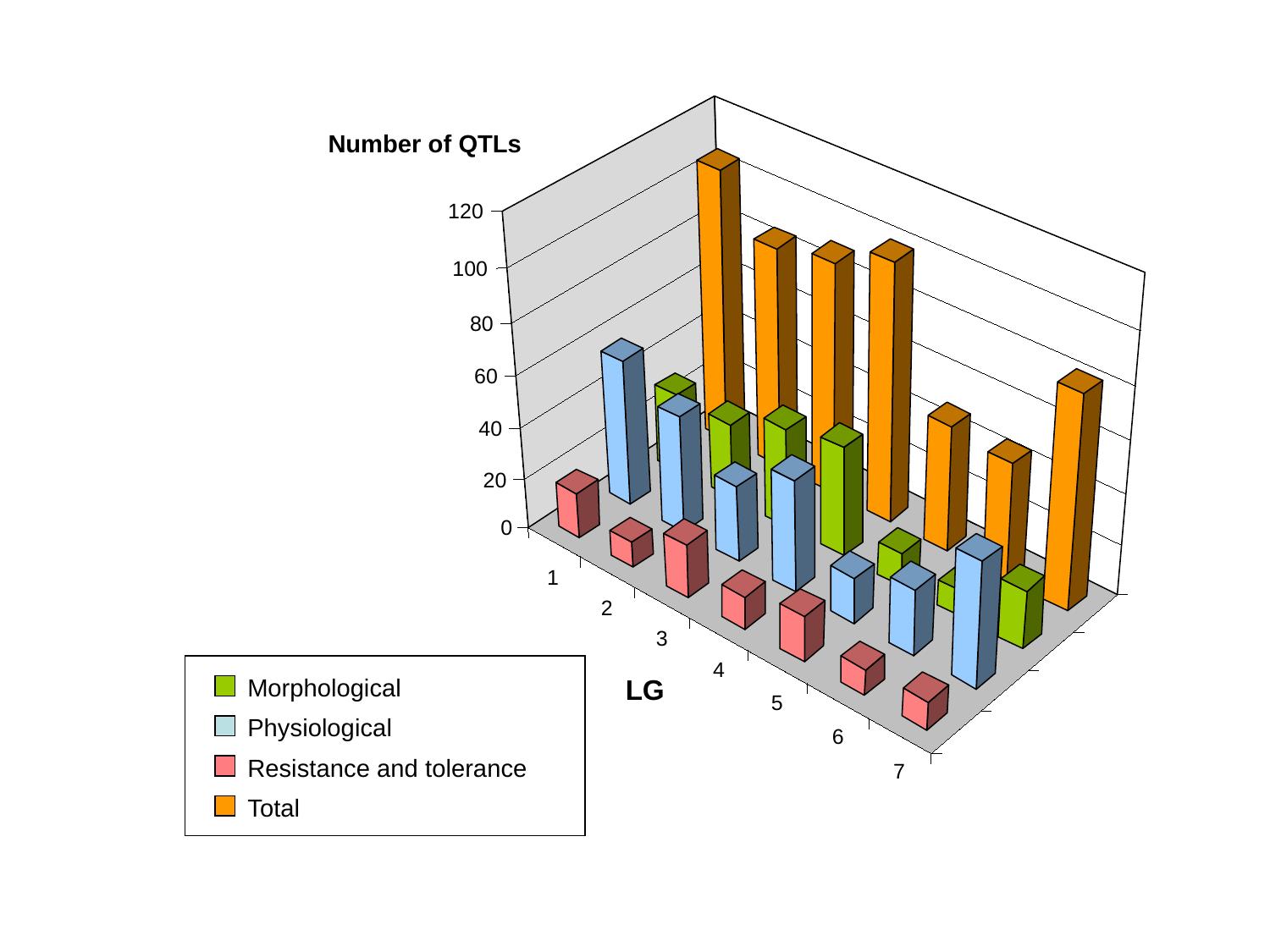

Number of QTLs
120
100
80
60
40
20
0
1
2
3
4
Morphological
LG
5
Physiological
6
Resistance and tolerance
7
Total

Supplement: Additional file 3 — Distribution of QTLs in each trait class on the seven perennial ryegrass LGs. [file 1471-2156-13-101-S3.ppt]
